# Potential factors associated with institutional childbirth among women in rural villages of Lao People’s Democratic Republic: a preliminary study

**DOI:** 10.1186/s12884-020-2776-7

**Published:** 2020-02-10

**Authors:** Sayaka Horiuchi, Bounthanome Nakdouangma, Thipphaphone Khongsavat, Shogo Kubota, Kazue Yamaoka

**Affiliations:** 1 Teikyo University Graduate School of Public Health, 2-11-1 Kaga, Itabashi, Tokyo, Japan; 2 Center for Birth Cohort Studies, University of Yamanashi, 1110, Shimokato, Chuo, Yamanashi, Japan; 3 Provincial Health Department, Sekong, Lao People’s Democratic Republic; 4 World Health Organization, Vientiane, Lao People’s Democratic Republic

**Keywords:** Institutional childbirth, Skilled birth attendant, Quality of antenatal care, Health education, Maternal knowledge, Rural village, Lao PDR

## Abstract

**Background:**

The provision of quality health services has been a global priority to reduce neonatal and maternal deaths. In Lao People’s Democratic Republic (Lao PDR), the coverage of institutional childbirth stayed at a low level regardless of a sharp increase in the coverage of antenatal care (ANC) and fee exemption. The aim of the present study was to preliminary explore factors associated with increased institutional childbirth and the association between ANC attendance and maternal knowledge among women in rural villages of Lao PDR.

**Methods:**

A secondary data analysis was conducted using data collected through a pilot survey in Sekong province in Lao PDR. The study participants were women with children under 5 years of age in villages within 10 km (km) from health centers staffed with skilled birth attendants. Data were collected via a face-to-face interview using a semi-structured questionnaire and were analysed using logistic regression models to estimate odds ratios (ORs) and their 95% confidence intervals (CIs) for having institutional childbirth in relation to potential factors.

**Results:**

A total of 302 women, 203 (67.2%) of whom gave birth at a health facility. 277 (91.7%) attended ANC at least once. Sixty-nine women (22.9%) had received no formal education, 272 (90.1%) were of an ethnic minority, 174 (57.6%) were unwaged and 99 (32.8%) lived more than 6 km from the nearest health facility. 51 (16.6%) did not know about birth complications at interview. Institutional childbirth was negatively associated with a lack of maternal knowledge about birth complications (OR, 0.27; 95% Cl, 0.14–0.54) after adjusting for covariates. Although there were few women who did not received ANC, the results suggested ANC might not be associated with maternal knowledge about birth complications (OR, 1.87; 95% Cl, 0.43–8.12).

**Conclusions:**

The present study suggests that maternal knowledge about birth complications is an important factor in increasing the institutional childbirth in rural villages of Lao PDR where majority of residents were ethnic minority. Improving quality of ANC and attitude among health care providers may be key to increasing health-seeking behavior. However, further research is needed to understand factors influencing choice of place of childbirth.

## Background

The reduction of neonatal and maternal deaths has been a global priority for decades. The Millennium Development Goals (MDGs) encouraged countries to increase their coverage of skilled birth attendance (SBA) at childbirth [1], and this strategy has passed to the Sustainable Development Goals (SDGs). Globally, the coverage of SBA at birth increased from 57 to 74% between 1990 and 2013 [2]. Despite this successful overall expansion of health care coverage, service utilization remains low in some regions and countries, and widening inequality in service usage has increasingly become a problem [3]. It has been reported that the disadvantaged women had limited access to health service [4–6]. Worldwide, 303,000 mothers died in 2015 and 2.5 million neonates died in 2017, mostly of preventable causes and due to poor quality maternal and neonatal care [7–9]. It remains on the worldwide agenda how best to improve effective coverage of health care services, to further reduce preventable deaths.

Lao People’s Democratic Republic (Lao PDR) had the highest neonatal and maternal mortality rates in the World Health Organization Western Pacific Region, at 27 neonatal deaths per 1000 live births in 2017 [8] and 197 maternal deaths per 100,000 live births in 2015 [10]. In Lao PDR, the proportion of pregnant women who had institutional childbirth still stayed at low level: 48.5% in 2015, regardless of an increase in the number of health facilities staffed with skilled birth attendants and a financial protection scheme for mothers and children through exemption from fees for health services and support for transportation and food [11]. On the other hand, there was a sharp increase in the percentage of women who received ANC at least once from 65.4% in 2013 to 91.0% in 2015 [11]. This suggested that mere deployment of skilled birth attendants and free service might not alone increase the utilization of institutional childbirth [12, 13] and implied a systematic problem in the health system that caused quite a few women to drop out from receiving continuous health care after they had accessed ANC. Reasons why institutional childbirth remained low regardless of relatively high ANC attendance in Lao PDR were not clear. Therefore, the present study attempted to look for preliminary information about how women’s socio-economic status, residential area, knowledge and ANC attendance were associated with institutional childbirth among women who lived in rural villages within 10 km (km) from a health center staffed with skilled birth attendants. The present study also tried to provide preliminary data about why women did not give birth at health facilities. We believe that the present study would provide a clue on the barriers of access to institutional childbirth and support further development of a larger research project to deeply understand and address the issues in Lao PDR.

## Methods

### Study design and setting

This is a secondary data analysis of a pilot survey that was conducted in the Lamam and Thateng districts of Sekong province between October and December 2014. The survey was conducted to provide preliminary information to consider questions regarding low institutional childbirth in the province that were raised by provincial health officials. The survey was led by the Maternal and Child Health unit of the Sekong Provincial Health Department (PHD) and the Japan International Cooperation Agency (JICA) project named “the project for strengthening integrated maternal, neonatal and child health services 2010-2015”. The project had permission to collect data in Lao PDR under an agreement with the Lao government including Sekong PHD [14].

Sekong province in southern Lao was one of the poorest provinces in the country, which had higher mortality of children under five and lower coverage of maternal and child health services compared to the national average as of 2012 [15]. Sekong province consists of four districts: Lamam, Thateng, Darkjung, and Kalum. By September 2014, district hospitals and most health centers in Lamam and Thateng had at least one skilled birth attendant. A skilled birth attendant was defined as a midwife, a nurse, physician, or medical assistant with midwifery skills, including a minimum capacity to provide basic emergency obstetric and new-born care [16]. Regardless of the deployment of skilled birth attendants, the proportion of births assisted at health facilities in the Lamam and Thateng districts were 42 and 26% respectively in 2014, according to data of the Sekong PHD. In each of the two districts, Lamam and Thateng, the survey selected the health center with the highest coverage of institutional childbirth and the health center with the lowest coverage of institutional childbirth, and then included all the villages within 10 km journey of those selected health centers.

### Study participants

The present study included women who participated in the preceding survey project and did not request an opt-out of the present study. The target of the preceding survey was women with children under 5 years of age at the time of interview. All women in the selected villages were recruited in the survey, and verbal consent was obtained from the chief of the village and from each woman individually prior to interview. Information and opt-out option of the present study was written in Lao language and sent out to village chiefs of all the target villages. Eventually, all participants of the survey were included in the present study. It was expected to include 300 women to examine factors associated with institutional childbirth as we estimated the effect size for the potential factors would be about 0.35 based on previous studies [17, 18].

### Data collection

In the survey, provincial officials working in the Maternal and Child Health unit conducted a face-to-face interview with each woman separately, using a semi-structured questionnaire (Additional file 1). The questionnaire was developed to collect data on socio-economic and demographic variables, ANC attendance during pregnancy, knowledge about birth complications, and potential barriers to institutional childbirth based on previous studies [17, 18]. To determine knowledge about birth complications, interviewees were asked whether they had heard about birth complications and whether they were concerned about the complications that could happen to them. Those who were concerned about the complications were considered to have a good understanding of birth complications. The present study did not consider home birth with SBA in primary analyses as it aimed to explore barriers of institutional childbirth compared to home birth without SBA. With regard to barriers to institutional childbirth, the interviewees chose all applicable answers from seven reasons presented in the questionnaire. The seven reasons were structured to cover physical accessibility, affordability, and acceptability [19], including distance from nearby health facilities, means of transportation to a health facility, affordability of care, and maternal perception of health care, which were considered to influence the maternal decision in seeking care [20]. These reasons were identified through literature review and opinions from health officials at the Maternal and Child Health unit of the Sekong PHD. The questionnaire was developed in English, translated into Lao and then translated back into English. A pre-test was performed for about 10 women in non-target villages within Sekong province and necessary adjustments were made to the questions to make them easier for villagers to understand. Interviewers asked women about their most recent childbirths. Interviewers were selected from the PHD instead health centers or district because health workers at the PHD had less relationship with the women interviewed. Provincial interviewers were accompanied by health workers from a district hospital and a health center and a member of a women’s union in the target village to easily identify households with potential interviewees. Only provincial interviewers met the target women at the interview, and district and health center staff did not directly talk with the interviewees. Provincial interviewers received a one-day training session on the final questionnaire.

### Data analysis

We examined the distribution of each variable among the study participants. We conducted univariate and multivariate analyses by using logistic regression models to estimate odds ratios (ORs) and their 95% confidence intervals (CIs) for institutional childbirth being related to each of the potential factors. The reasons why women did not give birth in health facility were summarized, with descriptions. We further analyzed the association between maternal knowledge about birth complications and ANC regardless of place of childbirth using a logistic regression model among women underwent institutional childbirth, home birth with SBA and home birth without SBA in order to explore whether ANC attendance could improve maternal knowledge in Lao PDR as ANC is a point of contact between women and the health system and is expected to deliver sufficient information to support women preparing for childbirth [21]. A stepwise method was used for variable selection with inclusion and exclusion criteria of 0.2 respectively. All the analyses were done with STATA/IC v14.0 software (StataCorp LLC, College Station, TX).

## Results

A total of 338 eligible women were interviewed in the target villages, and outcome data were available for 337 women. Regardless of the absence of civil registration, all women in the target area were included based on a report from a chief of each village. Among the 337 women, 203 women (60.2%) had undergone institutional childbirth, 35 (10.4%) had childbirth at home with SBA, and 99 (29.4%) had childbirth at home without SBA. As the objective of the present study was to find out why women did not have institutional childbirth, we excluded from further analysis those women who had childbirth at home with SBA.

Table 1 summarizes the characteristics of the 302 women included in the analyses. The maternal mean age at interview was 27.3 years (standard deviation (SD) 6.5 years). Sixty-nine women (22.9%) had received no formal education, 272 (90.1%) were of an ethnic minority, and 99 (32.8%) lived more than 6 km from the nearest health facility. At the time of interview, 174 (57.6%) of the households were unwaged. Of the 302 women, 277 (91.7%) received ANC at least once mainly at health facilities. Finally, 51 (16.6%) did not know about birth complications at interview.

**Table 1:** Institutional childbirth and characteristics of study participants (*N* = 302)

	Number (%)
Institutional childbirth	
Yes	203 (67.2)
No	99 (32.8)
Maternal age (years), mean [SD]	27.3 [6.5]
Less than 20	35 (11.6)
20–24	73 (24.2)
25–29	81 (26.8)
30–34	65 (21.5)
35 and more	47 (15.6)
Missing	1 (0.3)
Maternal educational attainment	
Secondary and above	82 (27.2)
Primary	151 (50.0)
No formal education	69 (22.9)
Work of women and house owner	
At least one employed/self-employed	126 (41.7)
Both unemployed/unpaid family work	174 (57.6)
Missing	2 (0.7)
Ethnicity of women	
Lao	30 (9.9)
Minority	272 (90.1)
Distance from the nearest facility (km)	
Less than 1	109 (36.1)
1–5	94 (31.1)
6 and more	99 (32.8)
Number of children	
One or two	145 (48.0)
Three and more	157 (52.0)
Maternal knowledge about birth complications	
Know	244 (80.8)
Do not know	51 (16.9)
Missing	7 (2.3)
Antenatal care during pregnancy	
Received at least once	277 (91.7)
Never	10 (3.3)
Missing	15 (5.0)

*SD* Standard deviation

Table 2 summarizes the results of logistic regression analyses. In the univariate analysis, a lack of maternal knowledge about birth complications (OR, 0.24; 95% CI 0.13–0.45), the absence of maternal formal education (OR, 0.18; 95% CI, 0.08–0.38), having more than two children (OR, 0.41; 95% CI, 0.25–0.67), an absence of ANC during pregnancy (OR, 0.05; 95% CI, 0.01–0.39), and ethnic minority (OR, 0.29; 95% CI, 0.10–0.85) were significantly associated with a lower probability of having institutional childbirth. Women living farther from a health facility were less likely to have undergone institutional childbirth than those living within 1 km of a health facility (OR, 0.28; 95% CI, 0.15–0.55 for 1–5 km, and OR, 0.22; 95% CI, 0.12–0.43 for 6 km and more).

**Table 2:** Crude and adjusted odds ratios of having institutional childbirth related to factors of women

	Crude OR (95% CI) *N* = 302^a^	Adjusted OR (95% CI)^b^ *N* = 295
Maternal age (years)		
Less than 20	1.00	-^c^
20–24	1.40 (0.57–3.41)	
25–29	0.87 (0.37–2.03)	
30–34	1.03 (0.42–2.50)	
35 and more	0.52 (0.21–1.30)	
Maternal educational attainment		
Secondary and above	1.00	1.00
Primary	0.32 (0.16–0.64)	0.41 (0.19–0.89)
No formal education	0.18 (0.08–0.38)	0.24 (0.10–0.57)
Work of woman and house owner		
At least one self-employed/Employed	1.00	-^c^
Both Unemployed/Unpaid family work/	0.64 (0.39–1.05)	
Ethnicity of women		
Lao	1.00	-^c^
Minority	0.29 (0.10–0.85)	
Distance from the nearest facility (km)		
Less than 1	1.00	1.00
1–5	0.28 (0.15–0.55)	0.33 (0.16–0.68)
6 and more	0.22 (0.12–0.43)	0.27 (0.13–0.54)
Number of children		
One or two	1.00	1.00
Three and more	0.41 (0.25–0.67)	0.49 (0.28–0.86)
Maternal knowledge about birth complications		
Know	1.00	1.00
Do not know	0.24 (0.13–0.45)	0.27 (0.14–0.54)
Antenatal care during pregnancy		
Received at least once	1.00	- ^c^
Never	0.05 (0.01–0.39)	

*CI* confidence interval, *OR* odds ratio

^a^Excluded those with missing data in the Table 1

^b^Adjusted for all other variables in the table except maternal age, work of woman and house owner, ethnicity of women and antenatal care during pregnancy

^c^Variable omitted in a stepwise variable selection and not included in the multivariate analysis

After adjusting all covariates that were selected in the stepwise method, including maternal educational attainment, distance from the nearest health facility, and number of children, women’s lack of maternal knowledge about birth complications was still related to a lower probability of having institutional childbirth (adjusted OR, 0.27; 95% CI, 0.14–0.54) compared to their counterparts. Although the present study focused on women living in villages within 10 km from health facilities, distance was still strongly associated with institutional childbirth after adjusting covariates. Women living 1–5 km from a health facility were 67% less likely (adjusted OR, 3.44; 95% CI, 1.72–6.87) and women living 6–10 km from a health facility were 73% less likely (adjusted OR, 0.27; 95% CI, 0.13–0.54) to give birth in a health facility compared with those living within 1 km of a health facility. Ethnic minority was no longer associated with institutional childbirth after adjusting covariates and was not selected in the stepwise methods. ANC did not converge in the multivariable analysis due to the limited number of women who did not receive ANC, therefore, ANC was not selected in the stepwise methods either.

Table 3 summarizes the association between maternal knowledge and ANC among those who underwent institutional childbirth, home birth with SBA, and home birth without SBA. ANC was not related to maternal knowledge about birth complications in either the crude or adjusted models. In fact, 15.7% of those who had ANC at least once did not know about birth complications, regardless of the high uptake of ANC (Table 4).

**Table 3:** Odds ratios of lack of maternal knowledge about birth complications in relation to other factors

	Crude OR (95% CI) *N* = 328 ^a^	Adjusted OR (95% CI) ^b^ *N* = 309
Antenatal care during pregnancy		
Received at least once	1.00	1.00
Never	1.61 (0.43–6.09)	1.87 (0.43–8.12)
Maternal age (years)		
Less than 20	1.00	1.00
20–24	0.61 (0.21–1.76)	0.66 (0.22–1.97)
25–29	1.22 (0.46–3.20)	1.38 (0.45–4.24)
30–34	0.37 (0.11–1.19)	0.44 (0.11–1.78)
35 and more	1.76 (0.63–4.95)	1.51 (0.38–5.96)
Maternal educational attainment		
Secondary and above	1.00	1.00
Primary	1.43 (0.68–3.03)	1.35 (0.56–3.28)
No formal education	1.85 (0.80–4.28)	1.43 (0.53–3.84)
Work of woman and house owner		
At least one self-employed/Employed	1.00	1.00
Both Unemployed/Unpaid family work	1.18 (0.65–2.15)	1.37 (0.66–2.83)
Ethnicity of women		
Lao	1.00	1.00
Minority	1.62 (0.55–4.79)	1,28 (0.32–5.09)
Distance from the nearest facility (km)		
Less than 1	1.00	1.00
1–5	3.08 (1.37–6.94)	2.80 (1.21–6.49)
6 and more	3.85 (1.76–8.43)	3.72 (1.59–8.70)
Number of children		
One or two	1.00	1.00
Three and more	1.01 (0.57–1.80)	0.79 (0.34–1.82)

*CI* confidence interval, *OR* odds ratio

^a^all women who had institutional childbirth, home birth with and without SBA were included, missing data were excluded

^b^Adjusted for all other variables in the table

**Table 4:** Association between maternal knowledge about birth complications and antenatal care visit at least once

	Antenatal care during pregnancy (*N* = 313^a^)		*p*-value (chi2)
	Received at least once	Never	
Maternal knowledge about birth complications			
Know	253 (84.3)	10 (76.9)	0.475
Do not know	47 (15.7)	3 (23.1)	

Numbers are number (%)

^a^All women who had institutional childbirth, home birth with and without SBA were included. Missing data were excluded

In addition to the main findings, Table 5 summarizes why women had childbirth at home without SBA in preference to institutional childbirth. Among the 99 women who gave birth at home without SBA, about half had limited physical access to a health facility, having no means of transportation or anyone to accompany them. They reported they would have difficulty in finding someone to look after them before and during their stay at a health facility. On the other hand, one-third said they could not afford to use a health facility, regardless of the fee exemption for maternal and child services in Lao PDR. They also considered that institutional childbirth was more expensive than home birth because women and their families had to pay transportation and buy food and other daily essentials in a facility, which they did not have to pay for otherwise. None of the women who raised the subject of a financial barrier knew about the fee exemption. A quarter of women claimed that they did not want to stay at health facility mainly due to fear or feel of embarrassment for seeing health care providers, and all of them reported that they felt more comfortable staying at home with their families and relatives. They preferred home birth to institutional childbirth as long as they had no complications.

**Table 5:** Reasons why women had home birth without SBA (*N* = 99)

Reason	Number (%)
Physical accessibility	
Did not have any means of transportation	51 (51.5)
There was no one accompany her travel	48 (48.5)
A health facility was too far to reach	31 (31.3)
Affordability	
Could not afford institutional childbirth	30 (30.3)
Acceptability	
Did not want to stay at a health facility	28 (28.3)
Did not see an importance of institutional childbirth	20 (20.2)
Her family did not allow institutional childbirth	14 (14.1)

## Discussion

Although the present study focused on women living relatively close to health facilities, about 30% of the women gave birth at home without SBA. Maternal knowledge about birth complications was a factor strongly associated with an increased probability of having institutional childbirth, regardless of the women’s educational attainments, ethnicity and distance from health facilities as reported in multiple studies in several low- and middle-income countries across different regions [4, 5, 22]. On the other hand, what is notable in the present study was that most of the women who gave birth at home without SBA received ANC at least once during their pregnancy. Unlike previous studies that reported a positive correlation between ANC and SBA utilization [4, 5, 18, 23–25], the present study could not pursue analyses of association between ANC and institutional childbirth in multivariate analyses due to the limited number of women who did not receive ANC. The results suggest that the health system in the target area failed to provide continuous care for a woman after she accessed the system for the first time.

Interventions to encourage birth preparedness and complication readiness during pregnancy have been strongly recommended by the World Health Organization (WHO) to promote timely utilization of SBA at childbirth [21]. Birth preparedness can be encouraged by increased maternal knowledge about danger signs; therefore, appropriate health education and counselling during ANC are considered to be a key intervention [5, 6]. Moreover, better communication between health workers and mothers is believed to play an important role in building mutual trust, which is essential for acceptability [26]. Although there were few women who did not receive ANC, the present study suggested that there might be little association between maternal knowledge and ANC in Lao PDR. This would suggest that ANC did not function sufficiently well in providing information and promoting birth preparedness among women in Lao PDR. Moreover, in the present study, women who attended ANC were not informed about fee exemption for institutional childbirth. Indeed, the overall quality of ANC in Lao PDR was reportedly poor, especially in health centers. Health care providers were not well trained to provide health education and/or counselling, and the average consultation time at ANC was reported to be only 5 minutes [27, 28]. Additionally, most health care providers belong to the dominant ethnic group and have difficulty in communicating with minorities who speak languages other than Lao. It is necessary to increase awareness of the importance of health education and counselling among health care providers, and to strengthen their communication skills to enable them to convey essential information and help mothers-to-be and their families to decide on the place of birth and prepare accordingly.

Another remarkable finding of the present study was that a greater distance between a residential area and a health facility was strongly related to childbirth at home without SBA, even though the present study focused on women living near to health facilities (within 10 km). The results are in line with previous studies that highlighted distance as being an essential determinant of service utilization [19, 23, 29–31]. However, the present study adds that accessibility could still be an issue among people living near to health facilities when birth preparation was insufficient. Most women in the present study who gave birth at home without SBA answered that they had difficulty in finding transportation immediately after contraction started. Some had further difficulty in paying for the transportation, as facility-based childbirth is fully subsidized by the government but does not fully cover the transportation fee. These findings suggest that better preparedness and financial support for the indirect costs of health service could mitigate some of the barriers of access to care.

The present study also suggested that uncomfortable experience at health facility would be an important barrier of having institutional childbirth. Most women who had home birth without SBA reported they felt embarrassed when they saw health care providers. Moreover, some of them were afraid to see health care providers. It has been reported that respectful maternity care is important to encourage service utilization and improve maternal and child health outcomes [32]. Women living in a rural village, being an ethnic minority or being in a lower socio-economic status were at greater risk of having disrespect and abusive maternity care [33, 34]. Although we could not perform deep assessment on maternal experience regarding disrespectful care in Lao PDR, the results suggest a necessity of improving attitude of health care providers toward pregnant women. More research may be necessary to understand more about disrespectful care in Lao PDR.

The present study has several limitations. As the present study incorporated a cross-sectional design, it was difficult to examine the temporal relationship between institutional childbirth and other factors. Data collection was fully dependent on interviewees’ memories and no cross-validation with medical records was performed. Therefore, there was a risk of recall bias, especially in reasons for not giving birth in a health facility. A further limitation is that the survey was conducted by health office staff, and interviewees may have been reluctant to criticize the health service. Therefore, maternal experience of disrespectful and abusive care at a health facility might have been underreported. As the present study focused on maternal factors, it had limited data to evaluate readiness and adequacy of treatment at a health facility. Finally, the survey was limited to women living in villages close to health facilities. The study results might not be generalizable to women living in more remote areas. The same applies to seasonality. The survey was conducted during the dry season, but the influence of distance on institutional childbirth might change in the rainy season. Nonetheless, road conditions usually worsen during the rainy season, and we therefore believe our findings regarding distance would still be applicable for the rainy season.

## Conclusion

Maternal knowledge about birth complications was a critical factor in promoting institutional childbirth among women living in villages within 10 km from nearby health facilities in Lao PDR. Improving communication between women and health care providers and strengthening health education and counselling at ANC would be important in promoting birth preparedness and further increasing the proportion of births assisted at health facilities. In addition, the present study suggested that women living more than 1 km from a health facility had difficulty in finding transportation, even though the present study focused on those who lived near to (within 10 km of) health facilities. Healthcare providers could facilitate mothers-to-be and their families to plan the place of birth, timing, financing and means of transportation in advance. The government also needs to consider how to reduce the indirect costs of service when designing the national health insurance scheme and a fee exemption for maternal care. This preliminary information would inform a more comprehensive exploration of the enablers and barriers to accessing institutional childbirth in Lao PDR.

## Supplementary information

**Additional file 1.** Questionnaire.

## Data Availability

The raw datasets generated and/or analysed during the present study are not publicly available because JICA has allowed it to be used only for the author’s research. But summary tables are available from the corresponding author on reasonable request.

## Abbreviations

ANC 4+: Four or more antenatal care visits
ANC: Antenatal Care
CI: Confidence Interval
JICA: Japan International Cooperation Agency
MDGs: Millennium Development Goals
OR: Odds Ratio
PHD: Provincial Health Department
SBA: Skilled Birth Attendance
SDGs: Sustainable Development Goals
