# Supplementary material for: Potential factors associated with institutional childbirth among women in rural villages of Lao People’s Democratic Republic: a preliminary study
Source: BMC Pregnancy Childbirth. 2020 Feb 10;20:89. doi: 10.1186/s12884-020-2776-7 (PMC7011605; doi:10.1186/s12884-020-2776-7)
Supplement: Supplementary file 1 — Additional file 1. Questionnaire. [file 12884_2020_2776_MOESM1_ESM.docx]

**Appendix 2.** Questionnaire for pregnant women

Target: pregnant women and women with child under 5 year of age

Name of interviewer_____________________

Village________________; District_________________; Province____________________

1. Individual information:
2. Distance (house to facility)_______Km Your age______;

Your husband’s age_____

1. What is your ethnicity?

□1. Lowland ; minor ethnic (name)

□2. Midland ; minor ethnic (name)

□3. Highland ; minor ethnic (name)

□a.Alak □d.Te

□b.Katu □e.Jatong

□c.Talieng □f. Other(specify)

1. Married status

□1. Married

□2. Widowed

□3. Divorced

□4. Separated

□5. Others (Specify)

1. What is the main occupation of the household head? (main occupation: work more than 4 days a week)

□ 1. Farmer

□ 2. Merchant.

□ 3. Others (Specify)

1. What is your main occupation?

□ 1. Farmer

□ 2. Housewife

□ 3. Merchant

□ 4. Others (Specify)

1. What is your last educational status?

□ No education

□ Primary

□ Secondary

□ Higher

□ Others (Specify) years

1. What is your husband last educational status?

□ No education

□ Primary

□ Secondary

□ Higher

□ Others (Specify) years

The attitude of pregnant women towards delivery in the health facility

Q1. Where did you give birth last time? (Don’t ask this question to the first pregnant)

1. Hospital/health center
2. Private clinic
3. At home with SBA
4. At home without SBA
5. Other : Specify____________________

Q2. Why did you choose to deliver at home last time? (Ask villager each choice)

(Only for villagers who chose 3,4,5 in Q1)

1. I didn’t think it’s important to deliver at the facility　　　Yes / No
2. The health facility is far. 　　 　Yes / No
3. I had no means of transportation　　 Yes / No
4. There was no one who sent me to the facility　　　 Yes / No
5. I had no money to pay for the service Yes(Year of your last delivery _____)/ No
6. I didn’t want to come to the health facility　　　 Yes / No
7. the family didn’t agree to give birth at the facility　 Yes(Who? ) / No
8. others (_______________________________)

Q3. Why you didn’t want to come? (Ask villager each choice)

(Only for villagers chose 6 in Q2)

1. I was afraid health staffs　　　 Yes / No
2. Health staffs won’t provide efficient Yes / No

service even if patients visit the facility.

1. Health facility is not clean. 　　　 Yes / No
2. I’m shy to have checked by health staffs　　　 Yes / No
3. Delivery at home is more comfortable. 　　　 Yes / No
4. others (_______________________________)

Q4. Where are you going to give birth for next time?

1. Hospital/health center
2. Private clinic
3. At home with SBA
4. At home without SBA
5. Other : Specify____________________

Q5. Why do you want to give birth at the facility next time? (Ask villager each choice)

(Only villagers who answered 1 or 2 in Q4)

1. I’m afraid to deliver at home. Yes / No
2. There was a problem in the previous delivery. Yes / No
3. Health staffs recommended me to deliver at the facility. Yes / No
4. We have moved to the nearer place to the facility Yes / No
5. I feel safer to deliver at the facility Yes / No
6. Because it is free service. Yes / No
7. Others ( )

Q6. Why you won’t give birth at the hospital/health center next time? (Ask villager each choice)

(only villagers who answered 3, 4 or 5 in Q4)

1. I don’t think it’s important to deliver at the facility Yes / No
2. The health facility is far. Yes / No
3. I have no means of transportation Yes / No
4. I have no money to pay for the service Yes / No
5. I am afraid health staffs Yes / No
6. Health center staffs won’t provide efficient service even if patients visit the facility. Yes / No
7. I don’t want to stay at the facility. Yes Reason why ( ) / No
8. the family doesn’t agree to give birth at the facility Yes(who? ) / No
9. others (_______________________________)

Q7. Have you ever heard that childbirth can cause to bad health or death of a mother?

1. Yes. I’m afraid about that
2. Yes, but I don’t care
3. No.
